# A Novel Method Facilitating the Simple and Low-Cost Preparation of Human Osteochondral Slice Explants for Large-Scale Native Tissue Analysis

**DOI:** 10.3390/ijms22126394

**Published:** 2021-06-15

**Authors:** Jacob Spinnen, Lennard K. Shopperly, Carsten Rendenbach, Anja A. Kühl, Ufuk Sentürk, Daniel Kendoff, Shabnam Hemmati-Sadeghi, Michael Sittinger, Tilo Dehne

**Affiliations:** 1 Department of Rheumatology, Charité-Universitätsmedizin Berlin, Corporate Member of Freie Universität Berlin, Humboldt Universität zu Berlin, and Berlin Institute of Health, 10117 Berlin, Germany; lennard.shopperly@charite.de (L.K.S.); shabnam.hemmati-sadeghi@charite.de (S.H.-S.); michael.sittinger@charite.de (M.S.); tilo.dehne@charite.de (T.D.); 2 Department of Oral and Maxillofacial Surgery, Charité-Universitätsmedizin Berlin, Corporate Member of Freie Universität Berlin, Humboldt Universität zu Berlin, and Berlin Institute of Health, 13353 Berlin, Germany; carsten.rendenbach@charite.de; 3 iPATH Histopathology Core Unit, Charité-Universitätsmedizin Berlin, Corporate Member of Freie Universität Berlin, Humboldt Universität zu Berlin, and Berlin Institute of Health, 13353 Berlin, Germany; anja.kuehl@charite.de; 4 Department of Orthopedics, Charité-Universitätsmedizin Berlin, Corporate Member of Freie Universität Berlin, Humboldt Universität zu Berlin, and Berlin Institute of Health, 13353 Berlin, Germany; ufuk.sentuerk@charite.de; 5 Department of Orthopaedic Surgery, Helios Klinikum Berlin-Buch, 13125 Berlin, Germany; daniel.kendoff@helios-gesundheit.de

**Keywords:** osteoarthritis, osteochondral explant culture, joint modelling, pharmacological assay, native tissue analysis

## Abstract

For in vitro modeling of human joints, osteochondral explants represent an acceptable compromise between conventional cell culture and animal models. However, the scarcity of native human joint tissue poses a challenge for experiments requiring high numbers of samples and makes the method rather unsuitable for toxicity analyses and dosing studies. To scale their application, we developed a novel method that allows the preparation of up to 100 explant cultures from a single human sample with a simple setup. Explants were cultured for 21 days, stimulated with TNF-α or TGF-β3, and analyzed for cell viability, gene expression and histological changes. Tissue cell viability remained stable at >90% for three weeks. Proteoglycan levels and gene expression of *COL2A1*, *ACAN* and *COMP* were maintained for 14 days before decreasing. TNF-α and TGF-β3 caused dose-dependent changes in cartilage marker gene expression as early as 7 days. Histologically, cultures under TNF-α stimulation showed a 32% reduction in proteoglycans, detachment of collagen fibers and cell swelling after 7 days. In conclusion, thin osteochondral slice cultures behaved analogously to conventional punch explants despite cell stress exerted during fabrication. In pharmacological testing, both the shorter diffusion distance and the lack of need for serum in the culture suggest a positive effect on sensitivity. The ease of fabrication and the scalability of the sample number make this manufacturing method a promising platform for large-scale preclinical testing in joint research.

## 1. Introduction

Osteoarthritis (OA) is a condition involving the degeneration of articular cartilage, sclerosis of subchondral bone and chronic inflammation of the synovial membrane. To date, it is the main cause of physical disability worldwide [1,2]. Both the underlying pathomechanisms of OA as well as the mechanisms of physiological reorganization of cartilage tissue are poorly understood. Due to this lack of understanding, the treatment of such defects remains challenging. However, research in recent years has provided increasing evidence that cartilage cells have the general ability to regenerate if stimulated correctly. Limited traumatic cartilage defects can now be successfully treated and regenerated by autologous chondrocyte transplantation and specific cell-modulating substances. The emergence of growth factors as therapeutic agents is expected to further enable the regeneration of osteochondral tissue [3,4,5], increasing the need for an adequate platform to assess cartilage (repair) treatment strategies.

Traditionally, two-dimensional chondrocyte cultures in monolayer and OA animal models have been the main tools available for preclinical testing of the efficacy and side effects (ADME (absorption, distribution, metabolism, excretion) screening) of such substances. Unfortunately, the former often leads to limited insights due to the lack of representation of the complex tissue composition of the joint, while the latter, in addition to ethical aspects, entails high costs and time expenditure and is therefore usually only applied when a basic efficacy already seems very likely [6,7]. Recently, this spectrum has been expanded to include sophisticated 3D cultures and organ-on-chip applications. However, 3D cultures are either effortful to maintain or, in the case of joint-on-a-chip, not yet commercially available. Therefore, rapid, agile research and development of regenerative therapeutics for cartilage regeneration is limited to a certain extent [8,9,10].

A sustainable middle ground in joint modelling is the use of tissue explants. Explants are living, native and functional parts from organs, which are obtained from donor tissues or cadavers. Explants can accurately represent part of the tissue composition and architecture. Specifically, the tissue cells remain in their native extracellular matrix (ECM) configuration and questions regarding their response to biological stimuli can be answered much more reliably in the native situation than, for example, with monolayer cultures of only one cell type [11,12,13]. However, this modelling technique is severely limited by the availability of donor tissue. Employing the frequently used technique of vertical punching of the tissue and subsequential production of osteochondral punchings with diameters of at least 3 mm, only a very small number of explants can be produced from a tissue sample, depending on the tissue properties. Furthermore, the diffusion of nutrients and potentially interacting proteins is severely impeded by the amount of glycosaminoglycans in the tissue [14]. Hence, the explant thickness negatively correlates with information that can be obtained about the behavior of cells located deeper in the tissue.

To address this issue, we have developed a low-cost and uncomplex method to produce explant cultures using a thin-section approach. Similar to the live-slice technology known from neurophysiology, the method allows the production of vital tissue slices with a thickness of 500–800 µm [15]. This allows the production of up to 100 slice cultures from a single tissue sample for subsequential analysis of many different substances and concentrations. Furthermore, the thin tissue architecture is suitable for easy-to-maintain semi-static culture conditions, as the perfusion distance is sufficiently short to reliably guide both nutrients and any therapeutic agents through the tissue to the target cells.

This study aimed to analyze the suitability of osteochondral slices for osteochondral tissue modelling and pharmacological screening of biologically active substances. For this purpose, we obtained a large number of human slice cultures from surgically explanted tibial plateaus and analyzed the behavior of the tissue over 21 days at the histological, metabolic and transcriptional levels. Furthermore, we examined the reactivity of the embedded chondrocytes to the factors TNF-α and TGF-β3, known to affect cartilage matrix synthesis, to determine whether the tissue is in a sufficiently close state to that of the native situation to be used as a testing device [16,17].

## 2. Results

To develop a physiological model for osteochondral tissue which allows for quick handling and high-throughput applications with limited donor material at hand, we developed a method for the manufacturing of native osteochondral live slice cultures from human joint tissue. 500–800 µm thick osteochondral slice cultures were prepared from 23 different surgically explanted tibial plateaus using a custom-made microtome insert and then cultured in hanging inserts of 24-well plates. In this study, we assessed whether the explanted live slice cultures maintain their physiological properties such as long-term cell viability, gene expression, extracellular matrix and responsiveness towards biological stimuli.

### 2.1. Suitability for Slicing Varies with the Degree of Subchondral Bone Sclerosis

23 of 25 donors were suitable for the production of slice explants. The extent of subchondral sclerosis of the bone proved to be a decisive factor for the suitability of slice culture production. The brittleness of the bone increases sharply with the degree of sclerosis, leading to the subchondral cancellous bone not being cut smoothly by the impact of the blade but instead being crushed. Three punch cylinders were easily obtained from all other donors, from each of which 30–36 slice cultures could be safely produced. For reasons of logistical feasibility, we only cultivated the slice cultures from one punch at a time-from a technical perspective, the cultivation of 90–100 slice cultures per donor would have been possible.

### 2.2. Confocal Laser Scanning Microscopy Shows Highly Conserved Spatial Cell Order

**Figure 1:**
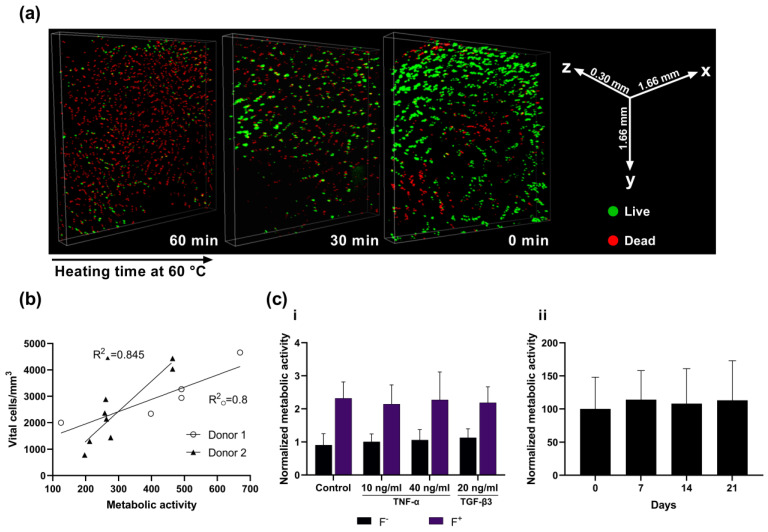
Viability analysis of osteochondral live slices. (**a**) Confocal laser scanning microscopy of slices at three weeks of culture following heating at 60 °C for 0, 30 or 60 min and live/dead staining with PI/FDA. Vital cells appear green, while dead cells appear red. (**b**) Correlation analysis of vital cell count as determined by CLSM and metabolic activity as determined by AlamarBlue resazurin assay. Two donors and a total of 13 slices with varying viability were analyzed and revealed correlation coefficients of 0.8 and 0.845. (**c**) Resazurin assay results with and without additional stimulation. i: Viability of slices on day 7 relative to day 0. Slices were stimulated with 10 or 40 ng/mL TNF-α or with 20 ng/mL TGF-β3, and either with or without FBS. ii: Viability of slices without FBS or other stimulating factors over 21 days, relative to day 0.

To validate the use of the resazurin assay and to evaluate the spatial distribution of living and dead cells, additional live/dead determination via CLSM was performed on 13 slices of two donors (7 and 8) after three weeks of culture. The slice explant cultures were thin enough to penetrate deep into the tissue layers with confocal laser microscopy and produce a three-dimensional image of the cell distribution- and vitality (3D rendered video in supplemental files). The analysis revealed >90% viability in non-heated slices, roughly 50% viability in slices that were heated for 30 min at 60 °C, and <10% chondrocyte viability after 60 min of heating (Figure 1a). Chondrocytes remained localized in their cartilage-typical, spatial alignment (column-like deep zone; pearl-bead structure in the mid-zone; tightly packed cells in the superficial zone, Figure 1a). No cell culture effects due to nutrition gradients were observed (e.g., no elongated cells in the peripheral tissue).

### 2.3. Resazurin Assay Correlates with Optically Determined Viability

To ensure that the resazurin assay sufficiently reflects viability and is not obstructed by the dense osteochondral ECM, samples of varying viability (obtained by heating at 60 °C) were analyzed with the resazurin metabolic assay and by live/dead determination via PI/FDA staining and CLSM analysis. The results of both methods were correlated, as shown by the correlation coefficient of R^2^ = 80–84.5% (Figure 1b). We regarded this as sufficient to assess the general viability state of the tissue culture. Further viability analyses of tissue cultures were therefore performed using the resazurin assay since it allows for progredient viability analysis of the same tissue slice in a non-destructible manner.

### 2.4. Cell Viability Is Maintained in F^−^ and Increased in F^+^ Groups over 21 Days

After 7 days, F^−^ cultured slices of the cytokine-stimulated groups exhibited either a slight increase or maintenance of viability as determined by the resazurin assay. Stimulation with TNF-α resulted in 101 ± 23% (10 ng/mL) and 106 ± 31% (40 ng/mL) compared to control, while TGF-β3 stimulated slices exhibited an increase to 113 ± 26%. Unstimulated slices showed a slight decrease to 91 ± 34%. No significant differences in viability from day 0 to day 7 as well as between the four different cytokine stimulation groups were observed. Addition of FBS to the culture medium resulted in significantly increased viability values in all stimulated groups (TNF-α: 214 ± 58% (low), 227 ± 84% (high); TGF-β3: 218 ± 48%) and the control (231 ± 50%) (Figure 1c(i)). During three-week culture without FBS viability values of 114 ± 43, 108 ± 52% and 113 ± 55% were observed after 7, 14 and 21 days, respectively. Statistical analysis revealed no significant differences in viability between the timepoints (Figure 1c(ii)).

### 2.5. Expression of Cartilage-Typical Markers ACAN and COL2A1 Is Maintained for 14 Days in Unstimulated Slice Culture

**Figure 2:**
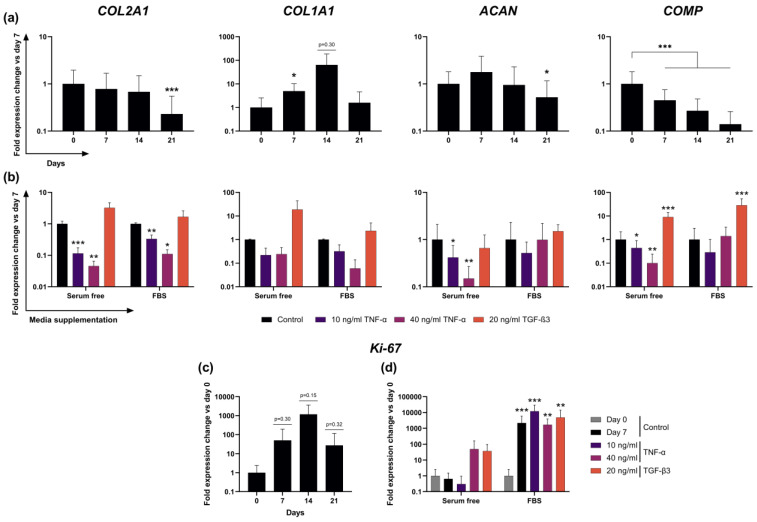
Gene expression analysis of live slice explants via RT-qPCR. (**a**) Gene expression of cartilage-typical markers *COL2A1*, *COL1A1*, *ACAN* and *COMP* in F^−^ cultured slices over three weeks (*n* = 12) relative to the day 0 value. (**b**) Gene expression of the same cartilage-typical markers as in A, after 7 days of stimulation with TNF-α or TGF-β3 and with or without FBS (*n* = 6) relative to the control on day 7. (**c**) Expression of proliferation marker *Ki-67* in F^−^ cultured slices over three weeks (*n* = 12) relative to the day 0 value and (**d**) after 7 days of stimulation with TNF-α or TGF-β3 and with or without FBS (*n* = 6), relative to the day 0 control to show the larger increase in the F^+^ group. Statistically significant differences denoted as * *p* < 0.05, ** *p* < 0.01, *** *p* < 0.001.

Expression of cartilage-relevant genes *COL2A1* and *ACAN* remained stable relative to their day 0 value for 14 days and declined significantly by day 21 in unstimulated slice cultures (no stimulating factors, no FBS) (*COL2A1*: 32 ± 23%, *p* < 0.001; *ACAN:* 51 ± 64%, *p* < 0.05; Figure 2a). Cartilage remodeling marker *COL1A1* showed a short increase after 7 days but returned to levels comparable to day 0 by day 21. Expression of *COMP* decreased sooner and significantly to 45 ± 30% after 7 days and further to 27 ± 21% and 14 ± 12% after 14 and 21 days (all *p*-values < 0.001).

### 2.6. Transcription of ECM Proteins Remains Highly Reactive to External Stimuli in F^−^ Culture

To test cell reactivity to external stimuli, we intended to induce measurable changes in chondrocyte gene expression. Slices were stimulated with TNF-α and TGF-β3 to either suppress or induce the expression of cartilage-relevant gene markers. Gene expression analysis showed differing results in the F^−^ and F^+^ stimulation groups. In the F^−^ group, stimulation with TNF-α resulted in significant and dose-dependent reductions of *COL2A1, ACAN* and *COMP* expression. Compared to the day 7 control, *COL2A1* expression exhibited levels of 11 ± 7% and 5 ± 5% in low and high dose TNF-α stimulations, respectively. *ACAN* expression also was significantly lower at 42 ± 33% and 15 ± 12%, while *COMP* expression dropped to 44 ± 48% and 10 ± 14%. Stimulation with TGF-β3 resulted in a 19 ± 25-fold higher *COL1A1* expression and 9 ± 5-fold higher *COMP* expression (Figure 2b). Expression of *MMP13* did not change significantly after incubation with TNF-α but was significantly lower after stimulation with TGF-β3 (6 ± 6%, Supplemental Figure S1). In the F^+^ group, stimulation with TNF-α resulted in a reduced *COL2A1* expression of 33 ± 11% in the lower concentration and 11 ± 4% in the high concentration compared to the control. Mean expression levels of *ACAN* and *COMP* in F^+^ also decreased after TNF-α exposure, although no coherent dose-dependent or significant effects could be observed. TGF-β3 stimulation resulted in a 29 ± 25-fold higher expression of *COMP* and a 2.4 ± 2.7-fold higher *COL1A1* expression (Figure 2b), as well as a decrease to 42 ± 40% of *MMP13* expression (Supplemental Figure S1).

### 2.7. Addition of FBS to Culture Media Causes Dominant Effects on Proliferation Marker Ki-67 Expression

Expression of *Ki-67* as a marker for cell proliferation in serum-free culture varied strongly over three weeks and between donors, as shown by the very high standard deviations and surge to a >1000-fold increase on day 14 before it returned to a 28 ± 90-fold higher level compared to day 0 on day 21 (Figure 2c). Even though the average increase in *Ki-67* expression from day 0 to 7 was substantial, presumably because of the low absolute initial expression of *Ki-67* at day 0, very high variability between donors was observed and led to statistically insignificant results. Stimulation with low concentration TNF-α in F^−^ groups led to 31 ± 65% of day 0 expression, while unexpectedly higher concentrated TNF-α resulted in a 50 ± 112-fold increase. TGF-β3 stimulation revealed a 37 ± 60-fold increase in expression after 7 days compared to day 0 control (*p* = 0.06). In the F^+^ group, however, all 3 stimulation groups and the control exhibited statistically significant changes between day 0 and 7, reaching fold-changes compared to day 0 of 12,045 ± 17,483 (TNF-α low), 1686 ± 2180 (TNF-α high) and 4889 ± 9110 (TGF-β3). No statistically significant differences were detected among the F^+^ cultured stimulation groups or between either of the groups and the day 7 negative control (Figure 2d).

### 2.8. Tissue Slices React to TNF-α Stimulation with Observable Remodeling of ECM and Cellular Swelling

**Figure 3:**
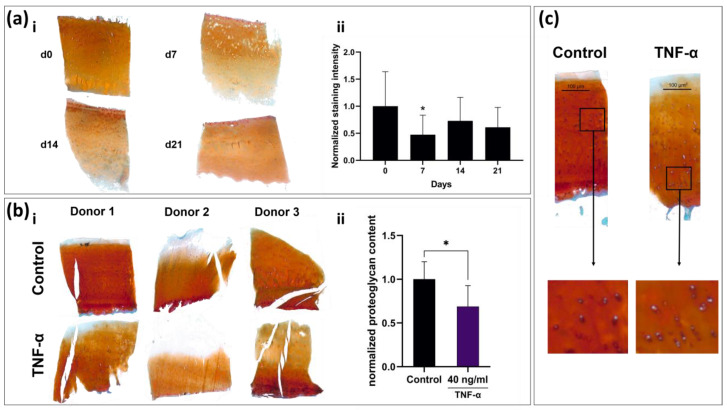
Histomorphometrical and histological analysis of live slices. (**a**) Safranin-O-stained slices of one donor (i) and histomorphometrical analysis (ii) over three weeks. (**b**) Comparison of staining intensity in TNF-α-stimulated and non-stimulated slices. Stimulated slices (three exemplary donors shown in (i)) show a mean reduction in red intensity of 32% (ii, *n* = 6). (**c**) Representative picture of TNF-α-treated slice and control. TNF-α-treated slices show less intense Safranin-O staining, loosened matrix structure and swollen cells in the mid-zone of the cartilage. Statistically significant differences denoted as * *p* < 0.05.

To analyze whether native slice cultures retain their tissue-specific histological composition or whether they can respond to molecular stimuli with remodeling of the ECM, the slices of long-term culture and those of TNF-α-stimulated cultures were stained with Safranin-O, because TNF-α is an important inducer of matrix degeneration processes in cartilage. The proteoglycan contents of non-stimulated, F^−^ cultured slices on days 0, 7, 14 and 21 (Figure 3a), as well as TNF-α-stimulated slices and negative controls on days 0 and 7 (Figure 3b), were quantified histomorphometrically. Even though a statistically significant decrease in proteoglycan content (*p* < 0.05) compared to day 0 was observed on day 7 in the long-term setup (Figure 3a(ii)), the mean proteoglycan content overall was maintained, as on days 14 and 21 it did not significantly differ from day 0. Proteoglycan content in the TNF-α-treated group was significantly lower than in the untreated control on day 7 (68 ± 24%; *p* < 0.05; Figure 3b(ii)). Corresponding to the changes in gene expression, TNF-α-stimulated slices exhibited a strongly altered histomorphology. Intra-cartilage fibers appeared less dense, while chondrocyte diameter and apparent volume increased (Figure 3c).

## 3. Discussion

The herein presented cost- and time-effective setup of surgical punch, 3D-printed microtome insert and rotary microtome is a reliable method to produce up to 100 explant cultures from a single human tissue sample. Critical to the usability of these thin osteochondral slice cultures is the long-term preservation of native tissue configuration and cell vitality despite thermal and mechanical stress development during preparation on the microtome and exposure of cells to atmospheric oxygen levels. CLSM analysis showed high cell vitality in both superficial and deeper tissue layers. Therefore, the slice thickness of 500 µm appears to be sufficient for cell protection within the tissue while ensuring adequate nutrient perfusion through motion-assisted diffusion. Furthermore, CLSM shows preservation of cartilage-specific cell architecture within the tissue even after three weeks of culture. Cartilage is divided into three distinct cellular zones: The superficial zone (densely packed with spindle-shaped cells), the middle zone (pearl cord-like alignment) and the deep zone (columnar chondrocyte alignment). Maintenance of the cellular zones depended on nutrient supply and physiological cartilage infrastructure, suggesting minimal cellular stress from malnutrition and/or hypoxia. However, as Secretan et al., already mentioned in their explant model, the disadvantage of the standard optical live/dead analysis of explants is the exclusive possibility of endpoint analysis and the uncertainty about apoptotic cells that may have been cleared in the meantime [18]. Therefore, the high correlation between cell vitality as determined by CLSM and viability determined by resazurin assay enable the utilization of the inexpensive and easy-to-handle resazurin assay for multi-point analyses in this culture form. This is particularly advantageous for high sample quantities, which can be created by this cutting method. Long-term analysis of cell viability revealed very stable viability values over 21 days with only minor fluctuations from the baseline. Interestingly, this stability was achieved in a regular cell culture medium without serum supplementation. Most explant cultures published to date use serum mixtures between 2–10% during cultivation [18,19,20]. While the addition of serum is a standard cell culture procedure, it is highly desirable, especially in light of pharmacological testing, if serum were not required as an undefined and potentially interacting, interfering or confounding factor for culture maintenance. This incentive is evident from the immense increase in absolute values and variation in viability values in the short-term culture with FBS addition, where toxicity effects would presumably not be visible due to the increased metabolic activity.

In terms of long-term stability, the slice explants provide similar characteristics to conventional punch explants. Bian et al. were able to demonstrate relative stability regarding the glycosaminoglycan content in serum-free cultivation of 3 mm thick explants over several weeks [21]. While we observed a significant decrease in the histomorphometrically determined Safranin-O staining intensity on day 7, it stabilized again on days 14 and 21. This could be due to an initial outflow of glycosaminoglycans at the cut edges of the cartilage, which account for a larger proportion of the total cartilage volume than in conventional punch explants. Regarding cartilage gene expression, the results are also similar to previously published analyses of explant gene expression. Different studies also described an initial increase of aggrecan followed by a decrease after 14 days and a decrease of the collagen type II expression of >50% after three weeks of cultivation following an initial stable phase [19,22]. *COMP* as a very sensitive marker for cartilage synthesis is the only gene in the analyzed panel to show a constant, almost linear decrease over time [23,24].

Stimulation of osteochondral slices with biological stimuli showed that chondrocytes in slice cultures responded adequately to external stimulation with catabolic molecules. TNF-α is known to be a proinflammatory cytokine in the joint and to induce cartilage matrix degradation both in vivo and in vitro [25,26], but other explant models either took significantly longer for a similarly strong reduction in matrix expression and degradation of proteoglycans or required additional catabolic stimuli such as oncostatin-M or interleukin-1β [11,27,28]. Here, the short perfusion distance between the medium and cells could be an advantage for pharmacological testing, since a shorter diffusion distance results in a higher and faster penetration by the corresponding factors than in thicker explant cultures. In addition to the decrease in anabolic biomarkers such as *COL2A1*, catabolic biomarkers such as *MMP13* also responded to external stimuli. Stimulation of the cultures with TGF-β3 resulted in close to complete suppression of *MMP13* expression more clearly observed in the serum free culture. Meanwhile, stimulation with TNF-α did not significantly increase *MMP13* expression. This suggests that due to the osteoarthritic damage and semi-static nature of the culture, only decreases catabolic marker genes are suitable as indicators of efficacy in pharmacological studies.

In studies aimed at analyzing pathological processes in cartilage or the long-term effects of an external stimulus rather than large-scale screening, it would also be beneficial to examine the distribution of the different collagen subtypes histologically, as these provide the most accurate information on cartilaginous remodeling processes.

Analogous to the viability measurement, it was also shown that when serum was added to the culture, the influences of the stimuli on the chondrocytes were significantly lower. In the serum-containing group, only the decrease of *COL2A1* expression following higher-dose TNF-α stimulation and the TGF-β3-dependent increase of *COMP* on day seven reached statistical significance compared to the control. Furthermore, analysis of the proliferation marker *Ki-67* showed that in the serum-containing group, all stimulated groups including the negative control showed a huge increase, whereas, in the F^−^ group, only TGF-β3 stimulation resulted in higher expression of *Ki-67*. This is further evidence of nonspecific responses by serum addition masking the specific effects of other stimuli, rendering it neither necessary nor advisable for pharmacological testing.

The method is limited by donor variability, the increased effort in handling, the use of priorly diseased tissue and its static nature. In comparison to conventional explants, which are merely punched out of the tissue specimen, slice cultures require several processing steps until successfully produced. Since the chondrocyte phenotype is strongly related to mechanical stimulation, several approaches are currently aiming to increase the dynamics and longevity of explant cultures by applying mechanical pressure [13,29,30,31]. This approach is more suitable for thicker, cylindrical explants and would, for the thin sections, be very difficult to implement. Therefore, the native tissue statues is inherently limited to a window of 3 to 4 weeks using this preparation method. This also limits the model in particular to the analysis of toxic-catabolic processes. Anabolic stimuli such as TGF-β3, while showing an increase in matrix protein expression at the transcriptional level, are associated with the simultaneous presence of mechanical stimuli [32]. A prior induction of catabolism would most likely be required to study the effects of an anabolic stimulus as previously described by Schlichting et al. [8,33]. Furthermore, the exact reproduction of slice thickness can be impaired by different qualities of bone density. Stronger bone calcification can make it necessary to cut the slice 100–200 µm thicker as the brittleness causes the bone to crush while being cut, resulting in unusable slices. Due to these slight variations in the cutting process, every donor must be related to itself when performing multipoint analysis. Therefore, the availability of a progredient analysis method like the resazurin assay is necessary to monitor individual slice viability. In accordance with this, a proteoglycan assay from the culture supernatants would also be conceivable for the analysis of the ECM. This could also provide continuous data from a slice culture instead of having to sacrifice the culture for histological endpoint analysis.

## 4. Materials and Methods

### 4.1. Sample Acquisition and Slice Production

**Figure 4:**
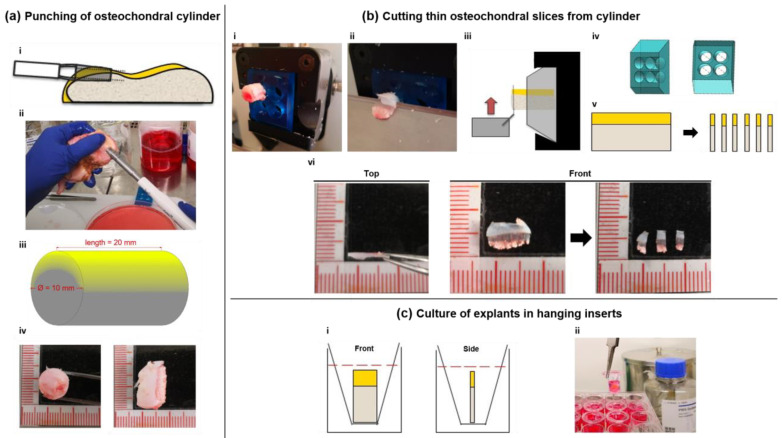
Fabrication and culture of osteochondral live slices. (**a**) Punching of Osteochondral cylinders. A 2 × 1 × 1 cm long osteochondral cylinder is punched out of the macroscopically unaffected area of a recently explanted human tibial plateau under sterile conditions using an orthopedic OATS^®^ Tissue Punch (i,ii). The resulting cylinder is shown schematically in iii, cartilaginous areas are highlighted in yellow. Front and side views of the cylinder are shown in iv. (**b**) Cutting of osteochondral slices from cylinder. The cylinder is inserted into a 3D-printed microtome insert (iv) with no additional fixation (i), and 500–800 µm thick cuts are cut out from the cylinder (ii). Fixation of the cylinder is shown schematically in iii. The resulting disc-shaped cuts (schematic: v) are cut into three parallelepipedal slices using a scalpel (vi). (**c**) Slices are immediately transferred to a hanging insert of a multi-well plate and covered in cell culture medium (i,ii). Plates are then placed on a horizontal shaker for culture at 37 °C and 5% CO_2_.

Whole tibial plateaus (TPs; *n* = 23; 13 female, 10 male, 61–89 years, ∅ 72 years) were acquired from patients undergoing knee arthroplasty comprising TP removal. The specimens were transported in serum-free cell culture medium (Dulbecco’s Modified Eagle Medium with 1 g/L glucose; 1% Penicillin/Streptomycin; 2% HEPES, Merck, Darmstadt Germany) to the laboratory with a maximum delay of 1 h. TPs were then immediately placed in Petri dishes filled with medium prewarmed to 37 °C under a laminar flow cabinet. After macroscopical inspection for an osteochondral site with a well-preserved cartilage-to-bone ratio (criteria: at least 1 mm high cartilage layer; no sclerosed subchondral bone below the cartilage), an orthopedic tissue punch (OATS^®^, Arthrex, Naples, FL, USA) press was used to create an osteochondral cylinder punch of 20 × 10 mm (Figure 4a). The cylinder was inserted into a custom 3D-printed microtome insert (see Supplemental Figure S2 for exact insert dimensions) and then cut into eight disc-shaped cuts with a thickness of 500–800 µm using a standard rotary microtome (Cut4060, MicroTec, Brixen, Italy). An 8 mm N35 microtome blade (FEATHER, Osaka, Japan) was used for cutting, which was exchanged after the preparation of 3 osteochondral cylinders. To produce a precise cut, the rotary handle was pulled downward at the point of maximum height in a powerful swing to exert maximum force on the cutting surface and thus prevent the bone from breaking. The cuts were then split into 3 cuboid-shaped slices using a scalpel, resulting in 24–36 individual slices per punch (Figure 4b). The remains of the cylinders were then discarded.

Two slices were immediately conserved either in 5% formaldehyde or in RNAlater^®^ (Thermo Fisher, Waltham, MA, USA), posing as day 0 samples for histological analysis and real-time quantitative polymerase chain reaction (RT-qPCR). Slices in RNAlater were subsequently stored at −80 °C. The remaining 22 slices were placed into hanging inserts (in a standing position with the cartilage facing up and leaning onto the upper rim of the inserts) of 24-well tissue culture plates with an 8 µm pore diameter (Transwell^®^, Corning, New York, NY, USA) for optimal perfusion. All treatments of the tissue were performed with sterile surgical gloves and instruments. Tissue culture plates were cultured at 37 °C and 5% CO_2_ on a horizontal shaker at 10 rpm (Figure 4c).

### 4.2. Maintenance and Stimulation

**Figure 5:**
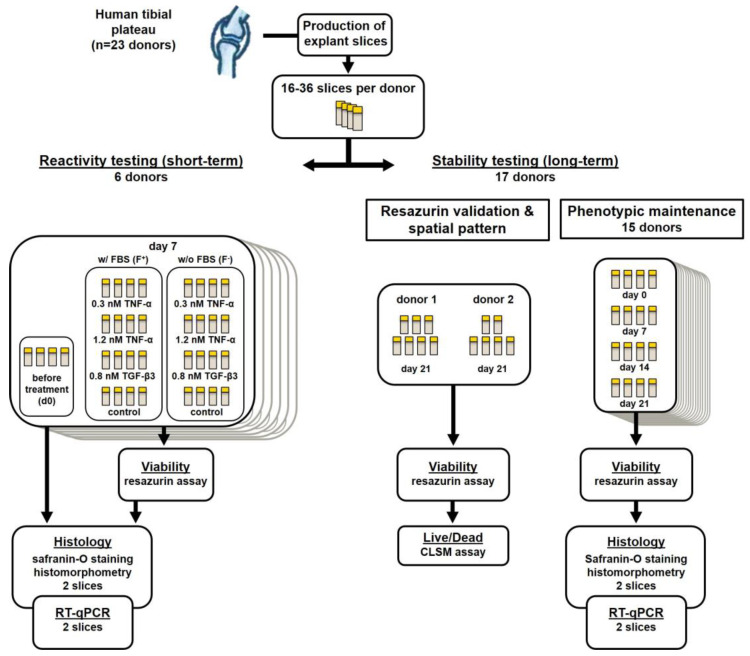
Flowchart depicting donor sample acquisition and distribution. Samples were obtained and then distributed for use in short-term (7 days, donors 1–6) stimulation experiments or long-term (21 days, donors 7–23) viability and gene expression experiments.

Slices of donors 1–6 were divided into a serum-free (F^−^) culture group and a serum-containing group (F^+^). The F^+^ group was cultured in the same medium as mentioned above with an additional 10% fetal bovine serum (FBS; Gibco, Thermo Fisher, Waltham, MA, USA). In both groups, four slices each were stimulated with either 0.3 nM (10 ng/mL) TNF-α, 1.2 nM (40 ng/mL) TNF-α or 0.8 nM (20 ng/mL) TGF-β3 (all Peprotech, Rocky Hill, NJ, USA) or no additives. Stimulating factors were pipetted with low-binding tips (Corning, New York, NY, USA) for minimal protein loss. Medium and stimulating factors were exchanged completely every three days. Analyses of slices for viability, gene expression and proteoglycan content were performed on days 0 and 7. Slices of the additional 17 donors were cultured without additional stimulating factors or FBS for 21 days in a large group viability and gene expression analysis (Figure 5). The medium was completely exchanged twice a week.

### 4.3. Resazurin Viability Analysis

Resazurin stock solution (AlamarBlue^®^, Thermo Fisher, Waltham, MA, USA) was diluted 1:10 with serum-free cell culture medium. Slices were taken out of their hanging insert, washed once with phosphate-buffered saline (PBS) and transferred into a well of a 96-well plate filled with 300 µL resazurin solution. Incubation of slices was performed for 2 h at 37 °C and 5% CO_2_. After the incubation period, duplicates of 100 µL of the resazurin supernatant of each well were transferred to a fresh 96-well-plate. Fluorescence was determined on a plate reader (Synergy, BioTek, Winooski, VT, USA), applying excitation/emission wavelengths of 540/590 nm. Slices were placed back into their hanging inserts after viability determination.

### 4.4. Confocal Laser Scanning Microscopy

To validate results from the resazurin assay and to evaluate the spatial distribution of living and dead cells, additional live/dead determination using confocal laser scanning microscopy (CLSM) was performed on 13 slices of two donors (7 and 8) after three weeks of culture. Prior to the resazurin assay, two slices were heated to 60 °C for 30 or 60 min to decrease intra-tissue cell viability to a minimum and obtain a portion of low-viability slices in comparison to the control. Subsequently, a resazurin assay was performed on all slices. Afterward, the slices were analyzed optically via live/dead staining with fluorescein diacetate (FDA) and propidium iodide (PI; both Sigma Aldrich, St. Louis, MO, USA). Slices were taken out of their hanging inserts, washed twice with PBS and incubated in 6 µg/mL FDA in PBS solution (25 min), followed by a 0.1 mg/mL PI in PBS solution (3 min). After a washing step, slices were immediately transferred into the microscopes incubating chamber. Tissue imaging was carried out with a Nikon Scanning Confocal A1Rsi+ (Nikon, Tokyo, Japan) at 37 °C in DMEM without phenol red (Thermo Fisher, Waltham, MA, USA). Excitation/Emission wavelengths were set at 488/590 nm for PI and 485/514 nm for FDA. The scanning area was set at 2.82 mm^2^. Pictures were stitched with a 20% overlap and their corresponding volume was determined using Nikon Capture NX-D software (version 1.6.2, Nikon, Tokyo, Japan). Z-stacking was performed with 10 µm spacing. Z-stacks were rendered into a 3D image with living cells depicted in green and dead cells in red. Living cells were counted using ImageJ software (version 1.8.0. [34]). For comparison of both methods, resazurin assay viability data was correlated to the number of living cells per mm^3^ sample using linear regression.

### 4.5. RNA Extraction and Real-Time Quantitative PCR

For RNA isolation, cartilage was carefully dissected from bony tissue, the resulting slices were soaked in RNAlater (Thermo Fisher, Waltham, MA, USA) and then stored at −80 °C overnight. The following day, the frozen tissue was transferred into liquid nitrogen and pulverized using a Biopulverizer (BioSpec, Bartlesville, OK, USA). Pulverized samples were transferred into a 90% TriReagent^®^, 10% 4-Bromo-2-chlorophenol solution (both Sigma-Aldrich, St. Louis, MO, USA) followed by centrifugation for 45 min at 13,000× *g*. The aqueous phase was collected, and nucleic acids were precipitated by the addition of an equal volume of ice-cold 70% isopropanol. After 30 min of incubation, precipitated nucleic acids were collected and resolved in RNA isolation buffer RLT (Qiagen, Hilden, Germany). Further purification was performed using a PicoPure™ Kit (Thermo Fisher, Waltham, MA, USA) according to the manufacturer’s instructions. The integrity and purity of RNA were analyzed using an Agilent Bioanalyzer 2100 (Agilent, Palo Alto, CA, USA); RNA concentration was assessed with a NanoDrop 1000 spectrophotometer (Thermo Fisher, Waltham, MA, USA). RNA was reversely transcribed using a cDNA synthesis kit (iScript™, BioRad, Hercules, CA, USA). RT-qPCR was performed in triplicates in 96-well plates (Becton Dickinson, Franklin Lakes, NJ, USA) on a Mastercycler^®^ ep gradient realplex (Eppendorf, Hamburg, Germany) using expression assays for TaqMan probes and primer sets (Thermo Fisher, Waltham, MA, USA; order no. in parentheses): collagen type II alpha 1 (*COL2A1*, Ss03373344_g1), collagen type I alpha 1 (*COL1A1*, Ss003373341_g1), aggrecan (*ACAN*, SS03373387_S1), *Ki-67* (*MKI67*, qHsaCID0011882) and cartilage oligomeric matrix protein (*COMP*, Hs01572837_g1). Expression analysis for matrix metalloproteinase 13 (*MMP13*, Ss033733279_m1) was only performed on short-term cultured slices. Succinate dehydrogenase complex, subunit A (*SDHA*, Hs00188166_m1) was used as reference gene. Marker gene expression is given as fold change compared to *SHDA* or control sample expression applying the efficiency corrected ΔΔ-Ct method [35].

### 4.6. Histological Analysis

Slices of each donor were fixated overnight in 5% formaldehyde solution and subsequently decalcified for 21 days in Osteosoft^®^ solution (Merck, Darmstadt, Germany). Afterward, slices were frozen in liquid nitrogen and subsequently cut into 4 µm thin sections using a CM19000 cryotome (Leica, Wetzlar, Germany). For Safranin-O staining, sections were stained for 30 min with 0.7% Safranin-O in 66% ethanol, counterstaining was performed with 0.2% Fast Green in 0.3% acetic acid (all Thermo Fisher, Waltham, MA, USA) for 1 min. To document ECM formation or loss, sections were mounted on glass slides; stainings were inspected using an AX 10 light microscope (Zeiss, Jena, Germany) and documented with a ProgRes^®^ SpeedXT core 5 microscope-mounted camera system (JENOPTIK, Jena, Germany). The intensity of the Safranin-O staining is directly proportional to the glycosaminoglycan content of the tissue and was therefore analyzed employing a histomorphometrical approach as previously described [8]. Briefly, pictures were taken and all pixels in the areas of interest were valued in the RGB color mode with a tool based on Xcode (Apple, Sunnyvale, CA, USA). When the red value (R) multiplied by 2 was higher than the sum of the green (G) and blue (B) values, the pixel was counted as red. The intensity of each red pixel was calculated as follows: intensity = 2 × *R*-value − *G*-value − *B*-value. Values of the intensity ranged between 1 and 508, and reporting images depicting the intensity distribution were created (see Supplemental Figure S3). The mean intensity (sum of intensities/area of interest) was calculated from each image.

### 4.7. Statistical Analysis

The significance level of log10-transformed data was determined with the independent two-sample *t*-test statistics of the Excel 2013 software package (Microsoft, Redmond, WA, USA). Normal distribution was checked applying the Anderson−Darling test, and equal variance of compared sample groups was tested applying the *f*-test. In all groups, signals were normally distributed. If the equal variance test was passed, student’s *t*-test was used, if not Welch’s *t*-test was applied. *p*-values < 0.05 were considered significant.

## 5. Conclusions

Native human osteochondral live slice explants represent a valid culture alternative to conventional punch explants. Its strengths lie in the greatly increased availability of tissue samples, as the thin nature of the slices allows for up to 50–100 pieces to be prepared from a single sample, the ease of fabrication and the simplicity of the culture in hanging inserts. This enables high-throughput screening of three-dimensional tissue processes and reactions in the native state that would otherwise require weeks of preparation in a tissue-based 3D culture or even in an animal model. Our results have shown that despite the stress of sharp dissection, the tissue fully retains its microarchitecture, and the cells retain their tissue-specific phenotype as well as their ability to properly respond to various stimuli. They also suggest that the short perfusion distance between cells and medium could also have a beneficial effect on the response strength of the cells to test stimuli, as the factors reliably reach cells embedded in the tissue. In addition, it was shown that the use of serum for cell expansion/survival is redundant in this culture form, which otherwise could mask adverse effects on tissue in the context of toxicity and degeneration analyses.

In summary, this production method is very well suited to produce native slice explants, which offer an interesting alternative to conventional explant cultures, especially in terms of pharmacological testing.

## Data Availability

The data presented in this study are available on request from the corresponding author. The data are not publicly available due to the use of patient material and the protection of personal data.

## Supplementary Materials

The following are available online at https://www.mdpi.com/article/10.3390/ijms22126394/s1, Figure S1: *MMP13* gene expression analysis. Figure S2: technical details of custom-made microtome insert. Figure S3: examples of histomorphometrical analysis output.
