# Supplementary figures and images for: A Novel Method Facilitating the Simple and Low-Cost Preparation of Human Osteochondral Slice Explants for Large-Scale Native Tissue Analysis
Source: Int J Mol Sci. 2021 Jun 15;22(12):6394. doi: 10.3390/ijms22126394 (PMC8232634; doi:10.3390/ijms22126394)

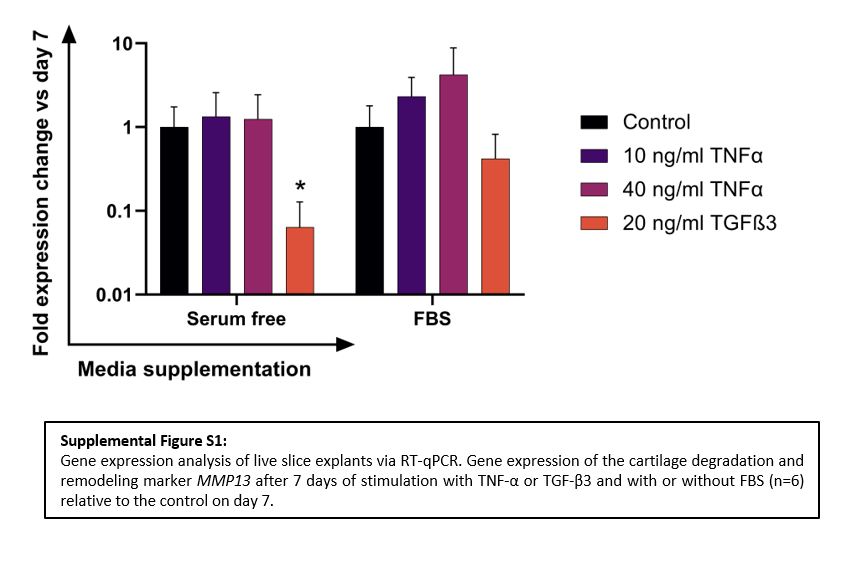

Supplement: Supplementary file 1 [file ijms-22-06394-s001.zip › SuppFig1.png]

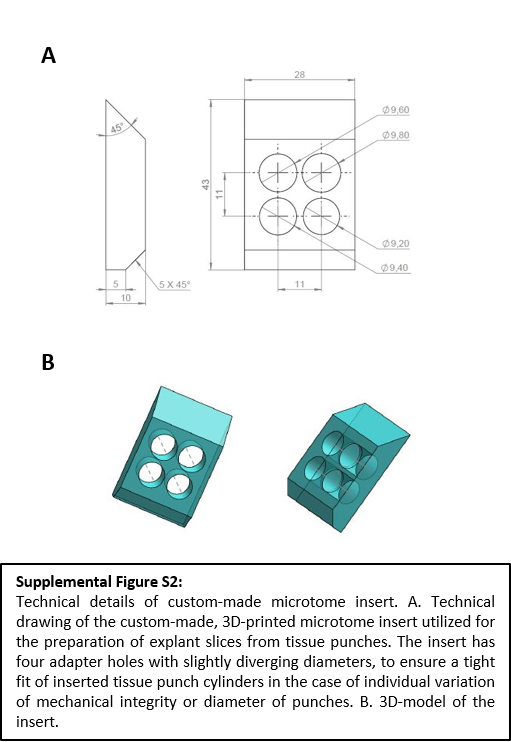

Supplement: Supplementary file 1 [file ijms-22-06394-s001.zip › SuppFig2.png]

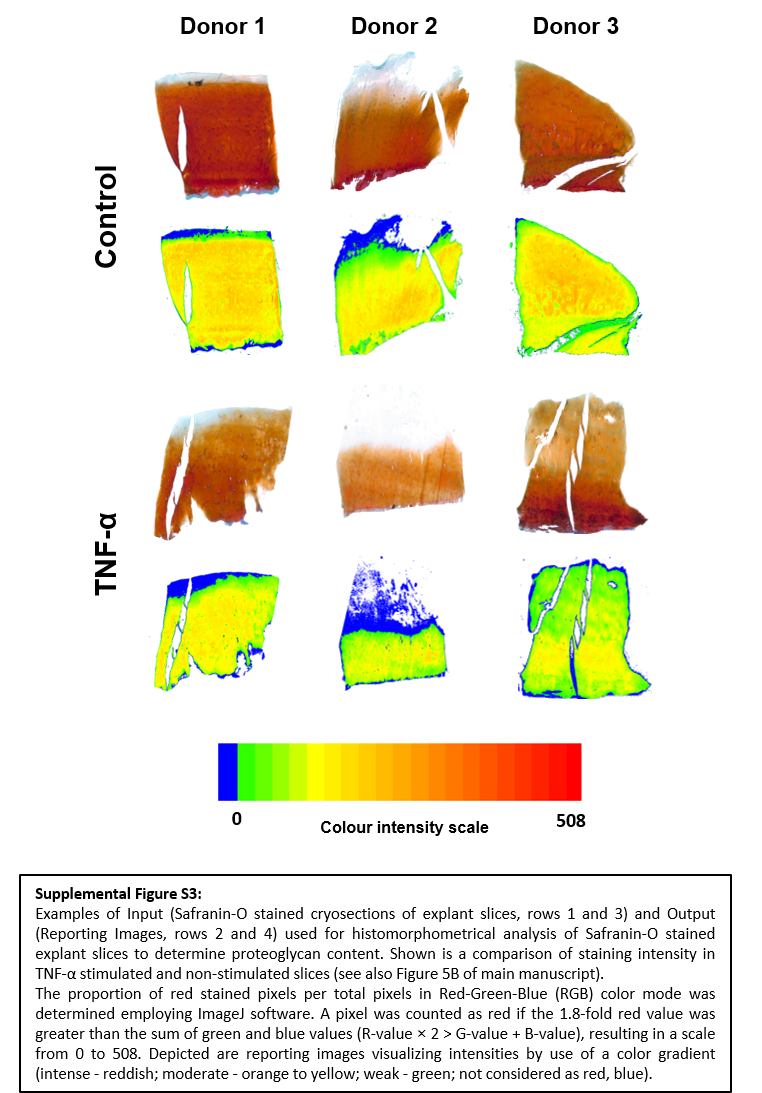

Supplement: Supplementary file 1 [file ijms-22-06394-s001.zip › SuppFig3.png]
